# The changing nature of life cycle assessment

**DOI:** 10.1016/j.biombioe.2015.04.024

**Published:** 2015-11

**Authors:** Marcelle C. McManus, Caroline M. Taylor

**Affiliations:** a Department of Mechanical Engineering, University of Bath, Bath BA2 7AY, UK; b Energy Biosciences Institute, University California Berkeley, 2151 Berkeley Way, Berkeley, CA 94720-5230, USA

**Keywords:** LCA, Bioenergy, Biofuels, Policy, Uncertainty, Sustainability

## Abstract

LCA has evolved from its origins in energy analysis in the 1960s and 70s into a wide ranging tool used to determine impacts of products or systems over several environmental and resource issues. The approach has become more prevalent in research, industry and policy. Its use continues to expand as it seeks to encompass impacts as diverse as resource accounting and social well being. Carbon policy for bioenergy has driven many of these changes.

Enabling assessment of complex issues over a life cycle basis is beneficial, but the process is sometimes difficult. LCA's use in framing is increasingly complex and more uncertain, and in some cases, irreconcilable. The charged environment surrounding biofuels and bioenergy exacerbates all of these. Reaching its full potential to help guide difficult policy discussions and emerging research involves successfully managing LCA's transition from attributional to consequential and from retrospective to prospective.

This paper examines LCA's on-going evolution and its use within bioenergy deployment. The management of methodological growth in the context of the unique challenges associated with bioenergy and biofuels is explored. Changes seen in bioenergy LCA will bleed into other LCA arenas, especially where it is important that a sustainable solution is chosen.

## Highlights

• Uncertainty needs to be addressed and accepted in LCA's development.
• Increased use of LCA is changing it, leading to questions about effectiveness.
• (Bio)energy LCA is a driver for many changes in LCA: challenges are seen here first.
• Both the driver and the force of the driver of LCA have changed (increased).

## 1 Introduction

Life cycle assessment (LCA) has become prevalent in research, industry and policy. From its origins in energy analysis in the 1960s and 70s, LCA has grown into a wide-ranging tool used to explore potential impacts to a range of environmental metrics and resource depletion. In that time, it has evolved rapidly from a tight, company based, attributional tool to one that is being more commonly used by policy makers and standards bodies for broad and interrelated effects beyond the project, for example, to help design large scale energy solutions. Whilst enabling complex issues to be assessed over a life cycle basis is beneficial in many respects, there are also difficulties associated with the expansion of LCA to meet this demand.

Bioenergy and sustainability policies across the globe are increasingly turning to life cycle assessment (LCA) to guide challenging decisions and select between technology paths, driven by carbon footprinting. Its use for bioenergy has made LCA mainstream, bringing with it all the benefits of a life cycle approach, but also the difficulties associated with applying it in practice. Although a conceptually simple tool, it can become convoluted in practice [1]. These difficulties are amplified as its use expands, along with the transition from attributional (aLCA) to consequential (cLCA) and from retrospective to prospective.

The public discourse around climate and the potential impact of biofuels has played a major, if indirect, role in the evolution of life cycle analysis, and in the development of consequential LCA. The public discourse surrounding biofuels, and to a lesser extent bioenergy as a whole, has been volatile since its inception and is highly polarized [2]. The popular press often contains overblown statements from both those opposed and those in favour, as well as the more measured voices (see, for example, [3], [4], [5], [6]). Anti-bioenergy rhetoric has been particularly vitriolic, in some specific cases for very good reason (for example, regarding oil palm on peat lands), which has come to represent all bioenergy for much of the public sphere [7], [8].

One particular aspect of the public debate has been closely tied to LCA's expansion, although the link is not immediately obvious: “food versus fuel” and the subsequent possibility of market-driven conversion of land to additional agriculture internationally. Popularization of the highly intuitive “food versus fuel” (more properly “feed versus fuel”) concept took hold strongly [7], [8], [9], [10]. This led to a spate of anti-biofuels articles in the press and NGO campaigns (e.g., [9]) in the late 2000s, and had a chilling effect on investment in R&D for (better) biofuels technologies [10]. It also influenced the policy discourse [11], [12] and resultant demands on accounting tools [2], [14] leading to some serious methodological developments and challenges.

These aspects of the public and political discourse have led to large changes in the nature of “life cycle assessment” and contributed to two somewhat distinct avenues of development and use, with a new one growing up alongside but seldom in concert with traditional LCA. One of the complexities associated with LCA is that it can be applied to a range of products or systems, over a product life cycle. The scope of analysis is set by defining a system boundary that explicitly identifies which aspects of the supply chain and processes are included, which allows for comparison of studies with the same boundary.

Traditionally LCA has been applied retrospectively to relatively contained (in terms of system boundaries) products or systems. This is now known as attributional LCA (aLCA). Recently there has been a move to apply life cycle assessments in larger scale decision contexts; effectively describing how environmental impacts might change in response to potential policy decisions (e.g., Ref. [13]). This is known as consequential LCA (cLCA). In effect, such use expands the system boundaries to include the activities contributing to any resultant changes. This adds to the complexity of the models and often means that a cLCA will include additional economic concepts such as marginal costs and data, and market mediated effects [15] and will look at impacts over a wider geographical and temporal range. Whilst aLCA traditionally focused on the use of linear, static models; cLCA cannot [15]. Nevertheless, a consistent approach for cLCA has not yet been established (see, e.g., Refs. [15], [16]).

The emergence of two branches of LCA with markedly different perspectives is one of the biggest challenges facing the LCA community, largely because their languages are widely disparate and the same terms (even “LCA”) may carry different meanings between them. The aLCA (original, old school, ISO governed), direct community and the cLCA (outcomes projected from taking a particular course) community do not overlap much, and language is nascent and inconsistent. The issue is inherently one of scale (and perspective – existing or concrete vs. projected). aLCA is micro scale and project specific; while cLCA, with its reliance on highly aggregated (by necessity) global economic models is macroscale. Joining the two areas is non-trivial. There are few examples of integrated a/c LCA teams, and unfortunately, there are few social sciences analyses of these communities. To the best of our knowledge, there has been only one, in which the authors were involved [17]. The issue of language (especially around what each group means by “uncertainty”) and mismatches in both discourse and goals between the communities are central themes that emerged. Taken together, these can make it challenging to discuss deficits and improvements or develop shared standards.

This paper examines the changing nature of LCA, focusing particularly on its use within bioenergy, which is driving many of these changes. Drawing on publication landscape analysis of the studies and method articles published in the peer-reviewed literature, we outline the stages of LCA development and the focus of work in the area. In the context of some of the demands LCA is faced with, the paper highlights methodological challenges and how effective the changes made to develop policy-relevant LCAs have been so far, with a particular example of land use change. The landscape analysis also suggests how LCA is likely to be used in the future.

## 2 History, trajectory and drivers in LCA development

**Fig. 1:**
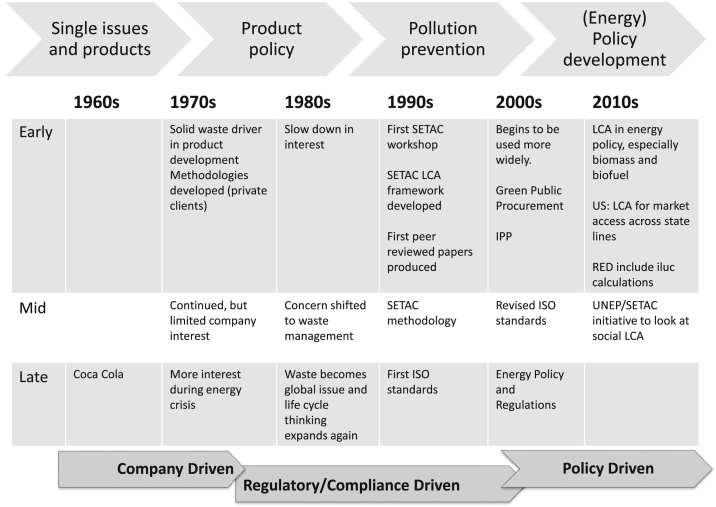
Trajectories and drivers in LCA development.

The use of LCA has increased rapidly since its conception, so that it is now a well-known and widely used tool across industry, academia and policy. From its start in the 1960s to wide-spread use in a little over 50 years, LCA has passed through three stages of development and adoption (Fig. 1), characterised by adoption drivers.

LCA emerged in the late 1960's as a tool developed and used by companies for resource management [18], [19]. It was predominantly single issue, such as waste, or single product based. In the US this was largely linked to Resource and Environmental Profile Analyses (REPAs) [20], and in the early 1970's solid waste management was a primary driver. Later in that decade the energy crisis drove companies to adopt an approach of energy management based on life cycle thinking (Fig. 1).

Many of these early LCAs, unfortunately, were not published as they were either commercially sensitive or internal company reports never intended for public distribution. One of the first of these encompassing a wider range of environmental impact analysis was produced for Coca Cola [21]. Though their proprietariness limited their availability, these initial early studies, and their clear value in design, set the scene for the wider ranging assessments that were to come after. They also helped to begin defining the methodology associated with determining impacts over a wide range of environmental issues. The first publication mentioning the term “life cycle assessment” and setting out a methodology, still generally used today, was in 1990 [22].

Towards the end of the 1980s and into the 90s, global environmental issues rose up the agenda [23], expanding from local and regional issues to encompass more international ones, including those garnering worldwide attention, such as ozone depletion and climate change. During this period the UN Conference on Environment and Development in Rio brought 150 nations together to set standards for global warming and individual nations and collectives (such as the EU) were beginning to take stock of the impacts of environmental pollution and hazards (e.g., [24]). The Brundtland [25] report, released by the UN in 1987, spurred increased interest in sustainability and sustainable development, and academic interest in LCA began to grow alongside, as it was seen to be an effective tool to calculate impact across a range of issues.

In the early 1990s the Society of Environmental Toxicology and Chemistry (SETAC) [26] standards were developed. These were based on the establishment of a retrospective tool to quantify the impacts of a particular product. At this stage the tool began to be used for regulatory purposes, but was still based on a retrospective approach. It was also associated with marketing, eco-labelling, packaging legislation and the suite of integrated product policy based (IPP) [27] regulations in the EU. The SETAC standards were adopted and amended to ISO standards in the late 1990s, forming the ISO 14040 series (ISO 14040–49 in 1997–2000). These were later revised in 2006 [28], [29].

Major policies incorporating LCA started with REPA [24], EPDs [30] and the IPP [27] for regulatory use, then surged with the major (federal & EU) level legislations that would govern state and member state energy: RED [31], EISA [32]. Out of these things such as RTFO [33], and RFS2/LCFS [34], [35] grew. LCA has expanded in both number and breadth and has become a wide ranging and far reaching environmental tool, even to the extent of setting policy [36], [37]. These policy-triggered jumps are tied to energy and bioenergy policies for GHG accounting. However, while this regulatory to policy trajectory was emerging, LCA was taking hold of the academic interest.

## 3 The development of LCA in the academic space

Academic interest in LCA began to grow at the same time that broader climate and sustainability issues were gaining attention and publications in the academic literature provide a valuable window into the expansion of the method.

**Fig. 2:**
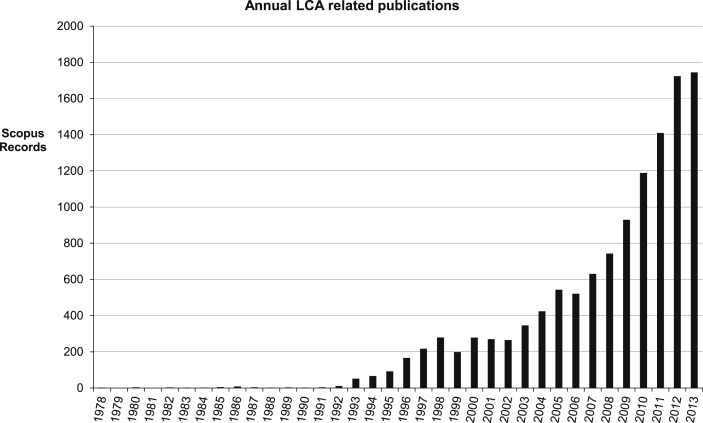
Annual LCA related publications.

The publication record reveals a rapidly expanding interest in assessment using LCA and in the method itself, taking hold within a remarkably short period. Fig. 2 shows the significant rise over recent years: 1992 is the first year with over ten publications in the area, rising above 1700 in 2013. The time course shows two distinct eras, an earlier, slower phase during which the concept was emerging, followed by a steep expansion reflecting recognition of the tool's use beyond compliance accounting.

### 3.1 Materials and methods

To identify and assess trends in the development of LCA as a tool and the evolution of its scope and methodologies, we assessed the publication landscape for LCA in the Scopus database from the first recorded publication in 1978 through 2013. Using Scopus advanced search options all publications on life cycle analysis in the academic literature in the assessment period were collected. A base search for all of the permutations on the phrase LCA in either title or abstract was then filtered using a series of iterative, compound Boolean searches to exclude records that are not related to life cycle assessment, using relevant environmental keywords selected from Scopus's list of keywords in the full search results and from terms identified in titles and abstracts. Errata were removed to avoid double counting. Finally, automated and direct comparison further reduced the publication set to a final set of records specific to LCA. These were then filtered for specific words in the titles and/or abstracts to assess shifts in publication and research trends. Searches were carried out in March and July 2014 and checked against each other, revealing no significant differences.

### 3.2 Early growth of LCA as regulatory LCA

**Fig. 3:**
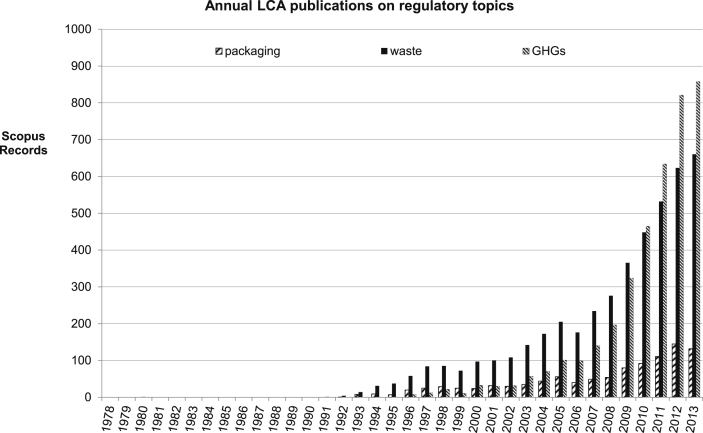
Annual LCA publications on regulatory topics.

**Fig. 4:**
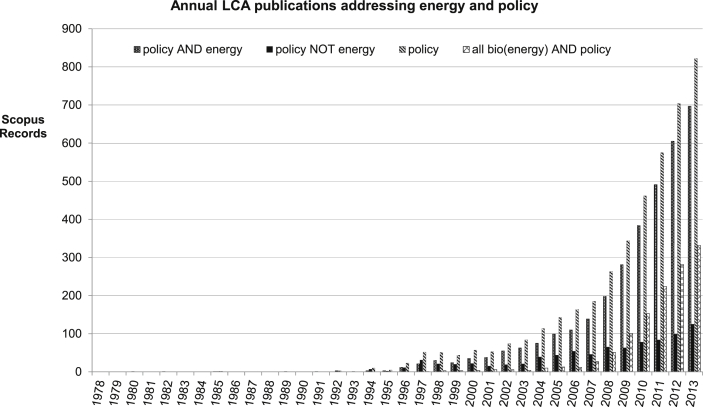
Annual LCA publications addressing energy and policy.

The turn of the millennium introduced an era of dramatic growth and change in LCA. The increase in LCA studies forms roughly two categories, between publications relating to regulatory topics, such as packaging, waste and greenhouse gases (Fig. 3), and the wider issues of policy (Fig. 4). Fig. 4 shows the beginning of more policy development using LCA, especially within the energy area, suggesting that energy a major driver for the expansion. This is borne out by the general discussion in this area. Interestingly, about half of the energy related policy LCAs relate to bioenergy, showing the special influence of bioenergy in this field.

### 3.3 Rapid growth of LCA/Policy LCA

Currently the predominant driver for much energy policy, and hence many energy related LCAs, is greenhouse gas accounting (e.g., RED [31], etc.). The change in drivers is fairly recent; GHGs overtook waste in the LCA publication record only in 2010 (Fig. 3). While recent, the speed of the shift is significant, clear in the volume of papers and studies produced in this period (from 33 in 2000 to 858 in 2013). The rate of LCA studies as a whole also shifts, from a slower, steadier accumulation relying mostly on additional waste and packaging analyses up to the early-mid 2000s, into a rapid explosion of papers dominated by GHG comparisons. The metric for emissions changed over the period, as well, to meet policy demands. From “emissions to air, water and soil”, air emissions evolved into aggregated GHG emissions, using the IPCC GWPs. The expansion to include other potentially related sources of GHG emissions, driven by the intersection of biofuels with agriculture, led to a still wider GHG metric. This suggests that not only has the driver for using LCA changed, so has the force of that driver.

### 3.4 Biofuels and bioenergy LCA

**Fig. 5:**
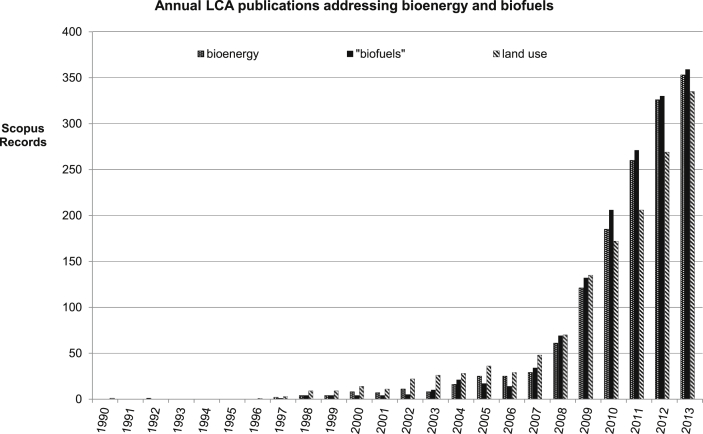
Annual LCA publications addressing bioenergy and biofuels.

The impact biofuels and bioenergy has had on the growing use of LCA from the early 1990s is clear from Fig. 5, with biofuels dominating bioenergy systems assessed. It is embedded in the period of rapid growth illustrated in Fig. 4. Work on biofuels technologies has expanded dramatically in the scientific literature. Alongside this has been a significant increase in number of LCA publications on biofuels and bioenergy (Fig. 5) that did much to drive the increasing number of LCA publications as a whole. Packaging grew very little. Waste, both waste processing systems and waste from other systems, accounts for about half the growth, and bioenergy (almost all of which are biofuels) the rest.

In the period, probably contributing to this sudden jump in growth rate, a number of simplified LCA tools became widely available, such as GREET [38] and GHGenius [39], enabling a wider audience to use the technique. Since the approach has a great deal of detail and complexity behind its simple result, this has introduced a new set of challenges, including unexpected – or unrecognized – difficulties in comparing studies or results.

The focus of work in GHG in the energy arena has provided a range of attributional type LCAs which illustrate the range of impacts across an array of energy technologies. Within these lie a collection of uncertainties (e.g., data gaps and varying scopes) [40] and sensitivities (e.g., missing or complex mechanisms), predominantly based on geographical and temporal differences, speed of technology change and improvement [41], and within the bioenergy arena, feedstock variation and land use change [42]. Despite these uncertainties and sensitivities these are essentially attributional LCAs. This increase in publications and interest in LCA reflects not only the increase in regulatory (i.e., compliance) ones that are mostly attributional LCAs (aLCA)), but also in the opening of a newer form incorporating potential effects of technology decisions.

**Fig. 6:**
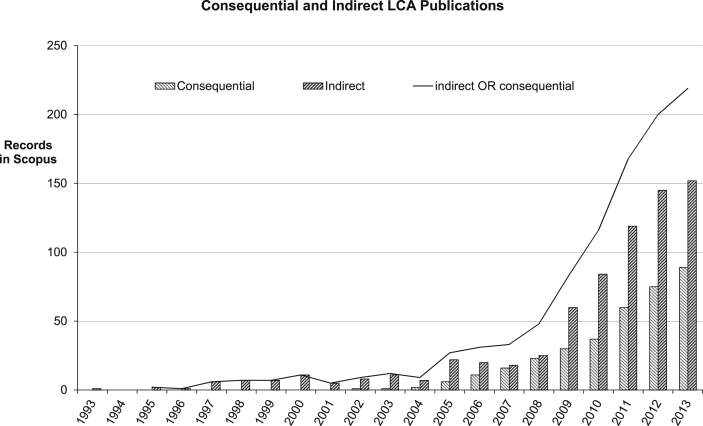
Annual LCA publications using or about consequential analysis.

The onset of expansion in the more consequential approach taken by some LCAs (cLCA), is clear in the growing use of indirect and/or consequential analyses shown in Fig. 6. Consequential LCA is broader, exploring not only the impacts of the production and use of a particular product in isolation, but the wider changes to the overall system that may arise from using that product, and often exclude the unchanged elements [43]. For example, a consequential analysis of a renewable energy technology might look at the impacts of the production, use and disposal of the technology, with increased emphasis on the impact of the offset of energy (or other substituted product) that would have been alternatively produced. It is essentially a policy tool, rather than a technology assessment tool, since policy decisions must take into account a broader range of factors [44]. Consequential analysis expands the system boundaries beyond those that have been traditionally set, and makes it an appealing tool for policy makers [31], [45], [46]. This emphasis can be seen in the increased use of the broad impact terms for indirect or consequential factors (Fig. 6), which are converging as the concept and label of cLCA gains popularity.

The expansion of the tool is not without problems. The systems LCA is asked to analyse are complex, and are becoming increasingly so. Many of the consequential LCAs to date have been developed from a series of attributional LCAs (e.g., a portfolio range of technologies working together or offsetting each other), but this is perhaps a simplification of the more complex reality, and some of these studies have been shown to give misleading results [47].

Bioenergy introduces many of these challenges into LCA, hence its impact as a driver for the recent evolution of LCA. Many of the bioenergy and biofuel technologies are nascent, or there are gaps in what is known or understood about their supply chains and mechanisms [48]. Consequential LCAs on bioenergy generally include data from several countries, industries and sectors, and will vary due to policy assumptions and market perturbations. In essence the system boundaries are being set on a global level, and there are problems with this, such as managing level of tractable detail, accounting for regional variation in substitutions or offsets or allocating impacts across multiple products (co-products) from shared production.

#### 3.4.1 Bioenergy's influences on LCA – the example of land use in LCA

The issue of land use (both direct and indirect) change in the bioenergy arena has large-scale policy impacts (e.g., RTFO [33], RED [31], EISA [32], LCFS [35], RSF2 [34]), through carbon payback and GHG accounting for market-mediated land use changes potentially associated with biomass use. Together these have had major impact on the nature of the emissions metric in LCA studies and in methods of calculation. In the more recent discourse a consequential approach to the analysis is more generally taken when discussing the impacts of land use change associated with bioenergy policies.

**Fig. 7:**
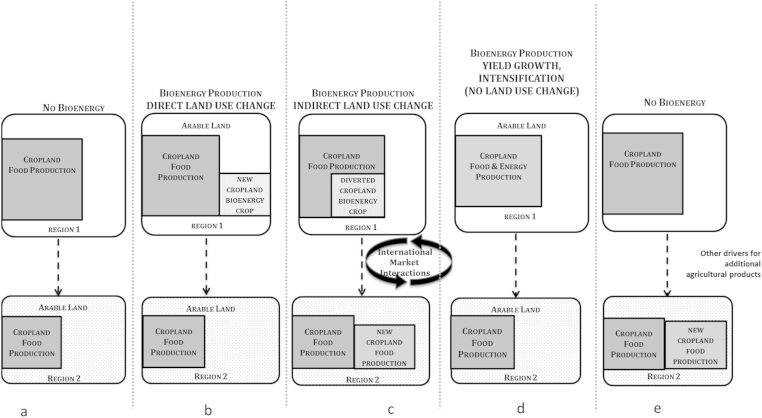
Direct and indirect land use change mechanisms.

Consequential LCA emerged in the wider debate with the concept of indirect land use change (ILUC) [49], which is based around global market responses to changes in demand for commodity grains, with effects felt in other areas, illustrated in Fig. 7. While direct land use change is prescriptive and fully causal for the particular project, indirect land use change is reflective of a change in state that could arise from the policy decision. The traditional inventory summation is insufficient for such analyses, which instead must use additional economic models and parameters. Panel 7a shows the static base case, assuming no change in demand from any factor, fuel or otherwise. Domestic production increases on lands from other uses, either other agricultural production or otherwise, would be Fig. 7b.

Fig. 7c illustrates the common form of the ILUC concept, in which the market drives conversion of land to new agricultural production. The quantity of land converted is estimated using various forms of equilibrium models for the global economy [50]. Because demand for grain as food is assumed to change little in response to price (i.e., has low elasticity value) [51], the idea follows that an area of land given over to bioenergy production gives rise to an equal amount of land being given over to food production elsewhere. Due to the global nature of trade this could be either close by (Fig. 7b) or in a completely different global region (Fig. 7c). The actual estimate of land conversion would depend on productivity and based on assumptions about the type of land converted. Environmental protections are generally assumed to be less rigorous in the external region. From this conversion, the GHG emissions are estimated with land-type specific emissions factors.

Estimates of the GHG impacts of ILUC can vary widely [52], [53]; for extremes ranges a particular biofuel's carbon footprint may span from below the fossil equivalent to over thousands of times worse [49]. Estimates vary depending on which model and economic database is used, productivity assumptions, management regime assumptions, emissions factors used, and scope of comparison, among other factors [54], [55]. Estimates are particularly sensitive to the starting state of land converted (the baseline) [56], [57]. The ranges of calculated values for ILUC reflect the system complexity as well as the method's maturity. As the models and data used to provide ILUC factors have improved so the GWP value associated with it has decreased across a given scenario. For example, for US corn ethanol the initial value was in 2008 was estimated at 104 g/MJ CO_2_eq and while estimated in 15 g/MJ CO_2_eq in 2012 [49]. Meta-analyses have illustrated this trend across a range of biofuel production scenarios and models [50], [52], [53], [55], [58]. As the number and range of scenarios assessed has grown, so has the range of estimated values, especially for analyses considering different management regimes (see, e.g., [59]), making robust cross-comparison more challenging.

**Fig. 8:**
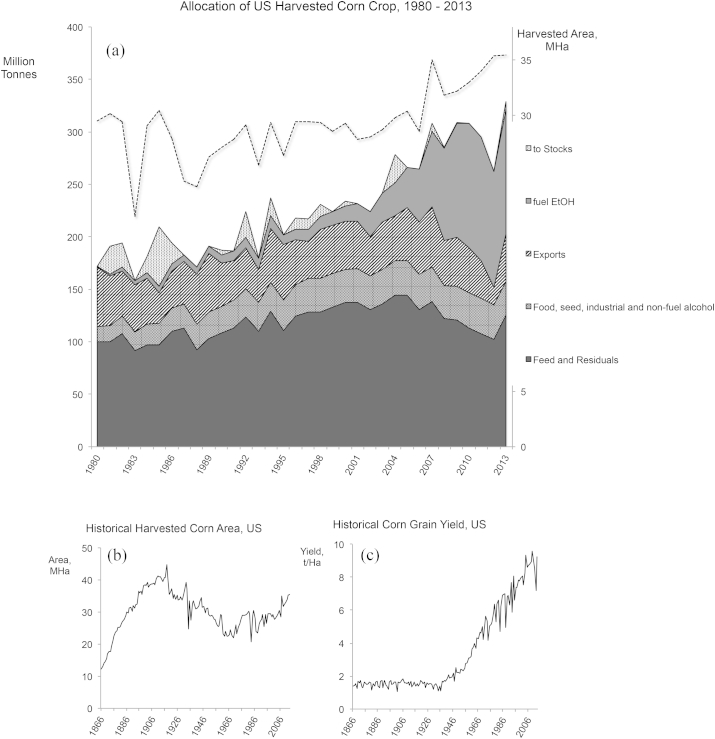
Production and Use Data for US corn grain. (a) Uses of annual corn crop in the US for fuel ethanol, livestock and other uses, with harvest area over the same period. Livestock feed is the largest use of grain, and does not include DDG(S) feed co-products from ethanol production. The fraction of the harvest to ethanol production has increased, along with overall production, and generally does not represent a decrease in absolute quantities to other uses. (b) Historical changes in corn grain yield in the US from 1866 to present and (c) Historical harvest area of corn. Yield gains in the modern era have more than compensated for expanded use across sectors and harvest areas have not yet returned to early 20th century levels, although land has been returning to production since the mid-1980's [48], [49].

The market interactions and assumptions that underlie calculations of possible indirect land use change are complex. Fig. 8 illustrates this with statistics for annual US corn grain production and allocations. The data suggest complexities that mean the simple land displacement model is insufficient. Panel 8a shows the US corn allocations since 1980 and the amount of land used in its production. The bulk of production is used for livestock feed (US corn is overwhelming grain corn, not sweet corn directly consumed by humans). Panel 8b shows land use for corn and 8c shows corn productivity over the same period.

Comparison to other shocks or rare events may provide some guidance for scenario assumptions. In many ways, the market behaviour in response to the 2012 US Midwestern drought mirrored the price spike of 2008 that generated such consternation and later retrenching [60], [61]. While total production was about 12% below normal, the drought did not overly affect food use (−2%); rather the impact was mostly to export (−53%) and livestock feed (largely met by DDGS), with stocks playing the buffering role for which they exist (here, ∼3% draw). Since exports and stocks are generally most influential on commodity grain prices [62], the intersection among food, fuel, nature and prices is a remarkably complex one [63]. Taken together, this means that the food versus fuel debate is an oversimplification (see Fig. 7c), and one that could lead to perverse consequences [64]. However, managing analyses of sufficient breadth and completeness to provide some estimate of long term impact is beset with difficulties, from data availability and uncertainty to sheer computational magnitude.

Large increases in crop productivity in the US since the 1800s (see Fig. 8c) have resulted in increasing corn production without extra land allocation. This productivity increase has been due to a number of advances; including strain selection, genetic modification (GM), and advanced crop protection and management. Fig. 7d shows this added complexity in terms of land use change. To add to the complexity, a variety of other agricultural products also influence international land use (Fig. 7e).

The static nature of the scenario analyses produces a common implicit assumption, that over the course of a field or project lifetime, there will be no improvements in management and/or productivity for other reasons. Indeed, many estimates assume current (and frequently current-worst-practice) productivity estimates over the full time horizon. The difference between that and a 10% increase in productivity is significant in most cases, for example reducing estimated carbon payback time for maize on degraded or croplands from about 75 years to just over 10 years [55], [56]. Even with the increased agricultural inputs associated with intensification and higher productivities, GHG mitigation benefits increased under these scenarios [65]. Incorporations of productivities are still static.

This is indicative of a more subtle, and more computationally challenging, assumption embedded in estimates of land availability: the amount of agriculturally-viable land is taken as finite. While essentially physically true (ignoring remediation), the amount of land *effectively available* is not fixed and instead depends on assumptions about management and productivity, among others, illustrated in panel 7d. Co-cropping or “land-sparing” agriculture [66], [67], [68] both result in increased effective land, although without policy supports may not increase conservation or carbon sequestration [69]. Such things are extremely challenging to include in a life cycle-based assessment, and rely on larger scale scenario comparison exercises. System boundary consistency here is crucial, because studies with differing system boundaries are not directly comparable.

The estimates of the impacts of the various bioenergy crops and fuels are therefore strongly sensitive to factors neither historically included nor readily tractable in an LCA, such as changes in productivity and other temporal issues. These illustrate that the implicit equivalence of fixed land amount for fixed grain (product) amount is incorrect. This poses some extremely daunting challenges for projecting (much less quantifying) impacts for systems that have an agricultural component. It also extends to other resource use, such as water. For setting long term policy, these are key issues for assessment methods to address because static analyses cannot adequately reflect the range of potential outcomes.

### 3.5 The rapidly evolving present of LCA

**Fig. 9:**
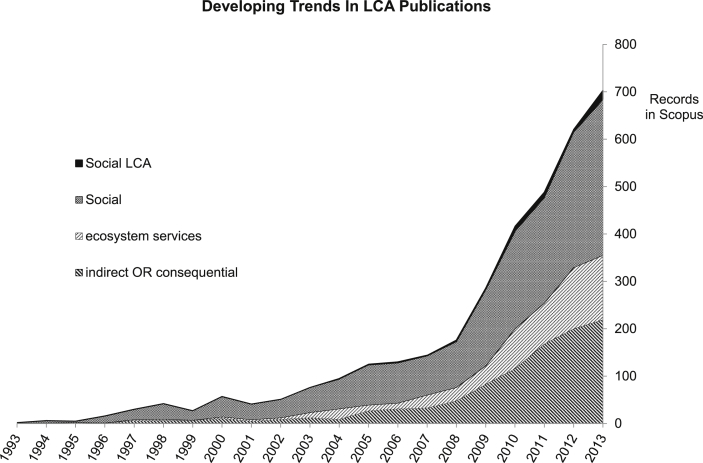
Developing trends in LCA metrics.

LCA has become a tool used to help drive and shape policy. Because of its history of efficacy, much of the emerging attempts to quantify such effects are appearing in LCA development and the published literature. Fig. 9 shows the expansion of LCA use in sustainability categories. Presently there is a drive towards ecosystem services, water and social impacts as well as the move from attributional to consequential LCA (Fig. 9). Although cLCA's expansion began around 2006, its uptake correlates with the growing emphasis on social and ecosystem service metrics that has started to expand from about 2010. Social aspects have expanded almost as rapidly as have indirect or consequential, and the beginnings of a formalisation of Social LCA can be seen just starting to emerge.

The emergence of consequential LCA illustrates the weight being placed on evaluating the impact of decisions on holistic sustainability. Social and economic effects are increasingly receiving the same weight as environmental ones. While social impacts have long been recognized as relevant for LCA (see, e.g., Ref. [70]), efforts to shape the tools to quantify such impacts have emerged with increasing interest in the last 5 years or so, from 2010 on, along with initiatives to set guidelines [71]. The trend to codify broader assessment approaches is not limited to social LCA. In this area, tools are much younger, though some approaches have started to emerge [72], [73], [74], along with increasing emphasis on integrated sustainability assessments [75].

Still too small to be seen in the aggregate publications, dynamic LCA is also beginning to develop as a means of incorporating temporal factors. Bioenergy introduces a number of time-dependent components to both the attributional and consequential analysis, some of which are handled in scenarios, others with ad hoc annualization, and still others not at all. These issues include, among others: field maturation and yield changes; technology and process changes over the length of the time horizon, which can be decades or centuries; market response times [76]; “carbon payback”; and the time separation between carbon uptake and release in combustion. The distinction between fossil and so-called biogenic carbon is a special case of the final item: the carbon released from fossil fuel is of prehistoric origin, while uptake and release of biogenic carbon are separated by as little as a year (annual crops) to many decades (woody residues). Management regimes also strongly influence the results and estimates of soil carbon storage [77]. This is of particular interest to bioenergy as biogenic carbon, and its storage, is considered as part of the life cycle of the fuel.

Temporal modelling is not standard in LCA, and temporal issues are handled on an ad hoc basis. In energy research, temporal issues are often considered as part of scenarios, or to accommodate changes in future electricity grid mixes (e.g., Refs. [78], [79]). Currently time issues are generally included by comparison between results between time points or linear averaging over the project timeline. So-called dynamic LCA is emerging to allow for time-dependent terms. However the level of inclusion of temporal issues even within a dynamic LCA varies, with some including temporal issues in the inventory [80], and some on the impact assessment [81]. Discussion and development of temporal factors in LCA is active [82], but routines have not yet made their way into common use.

## 4 The future of LCA and emerging challenges

Attributional LCA assesses technology and process for particular bioenergy projects or proposed technologies. Consequential LCA takes into consideration the systemic responses to bioenergy expansion, e.g. land use change and food crops. But LCAs need to model displacement of alternative products as a dynamic response to market interactions as well as focusing on only one use of a crop or system. The same is true for ecosystem services. These are symptomatic of LCA's future. The normalization of the tool for policy is spawning new methodological structures, among them social LCA and hybrid approaches.

Isolated comparisons are of limited value in assessing potential impacts across any entire system, like global climate, but methods remain immature for such challenging analyses. The concept illustrated by ILUC - that of indirect and/or market- or otherwise mediated impacts - is a component of planning beyond the bioenergy discourse, but part of the wider discussion of land management. This is one of the examples of where issues found in the bioenergy LCA arena are shaping LCA in general. However, examples of land use illustrate that there is a far more complex interaction than is often presented [63], and ILUC is an area in which the capabilities of the modelling are rapidly outpacing the scientific understanding and experimental data. There are also substantial concerns the indirect land use approach is fundamentally flawed, for example Zilberman et al. [83] who state that ILUC is a “second best solution to a first class problem”.

Thus the expansion and evolution of LCA faces four main categories of developmental challenges: establishing mechanisms and models; gaps in data and knowledge; incorporating temporal and dynamic components; and comparability limitations derived from differing on scenarios and system boundaries.

Since the concept of aggregate consequences and potential impacts is integral to long-term strategic decision making, the cLCA approach will extend into other sectors. Some of these will still be linked through land use, such food products and alternative energy and electricity sources, but the principle applies particularly where there are intersections with a core shared resource, e.g. industrial production with minerals or water. A consistent approach is required across sectors, which starts with uniformity in system boundaries and the decision points in defining the scenarios that determine that system boundary. Indirect land use change in the consequential approach is one aspect, but other products or resources have other potential impacts as a consequence of a policy decision.

**Fig. 10:**
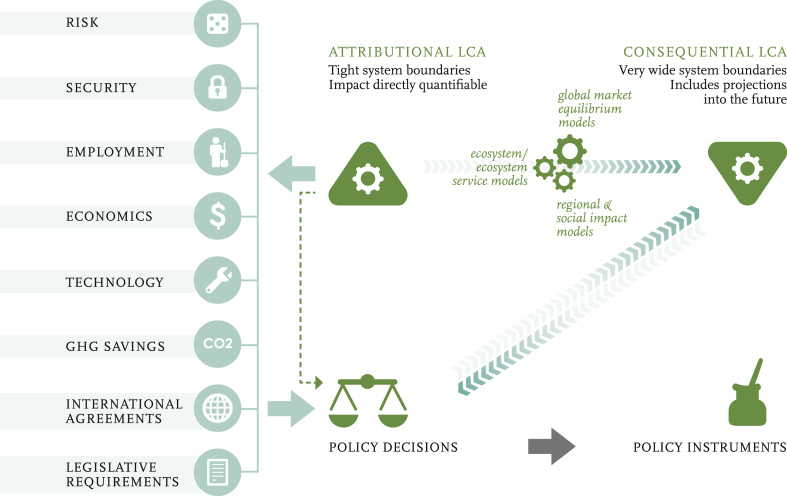
Illustration of the formative feedbacks among policy drivers and LCA. From Ref. [16].

Given the range of policy factors, and influences on stakeholder decisions [84], the future of LCA, or some similar tool or suite of tools, seems set to expand, and it is a transitional and exciting time for the approach [21], [85]. Fig. 10 illustrates the mediated feedbacks among methods and policy that are likely to inform development. Land use and carbon paybacks are used in this paper as an example of the issues described. But there are others, including the use of cLCA to explore impacts of changing energy mixes on carbon outputs due to changes in the grid [86], and the difficulties in associated modelling (e.g. offsetting simple marginal, dynamic marginal, or average grid mix). These offer similar challenges.

Effective policy guiding consequential LCAs would also therefore benefit from other tools, including and beyond economic equilibrium models [47], [87]. This idea that LCA should be used as part of a suite of tools is not new; academics and practitioners discussed it more than a decade ago (e.g., Ref. [88]), it is critical to wider sustainability policy planning. Indeed, Guineé et al. [21] propose that what is now needed is Life Cycle Sustainability Analysis (LCSA), which will comprise a suite of tools and multiple metrics required for a more comprehensive approach to impact assessment. This would set LCA amongst a suite of tools and enable it to develop more effectively to answer more specific questions [89].

## 5 Conclusions

Bioenergy has contributed to the fastest era of both change and use in LCA's short history. The pace of LCAs adoption has meant that it is sometimes used to answer questions it cannot, and this has given rise to questions about its effectiveness. There have been three phases in LCAs evolution thus far, traditionally based on a retrospective analysis, moving towards the more forward looking consequential analysis. Methods and data have not caught up with demand.

In order for LCA to develop effectively from a tight attributional tool to the wider reaching consequential tool that can be effectively used by and for policy makers investment is required from both the technical and the policy based community. Targeting that investment in three main areas can support the expansion of LCA and maintain or bolster credibility and reliability of the approach:

• *Creating fora for greater integration between the attributional and consequential communities, and with end user stakeholders to develop effective and objective tools*
• *Developing transparent mechanisms to convey uncertainty and comparability*
• *Data compilations/research to fill data gaps and research into and validation of feedback mechanisms in the methods.*

Life cycle assessment will only ever be as good as the data and assumptions it uses. It will also only ever be as good as the people who use the results, in that any results must be used in the context for which they were developed. To do otherwise will contribute to decreasing faith in the tool.

These issues are described in relation to bioenergy not only because bioenergy is an emerging energy source that is much assessed in terms of GHGs and environmental impact, but also because the complex global nature of agriculture and food and energy markets means that many of the issues described are emerging here. It is an early case in which the sectors are inseparable. During this period of change the links and connections between bioenergy LCA and policy are shaping each other. It is likely that the changes seen in LCA as a result of policy push will bleed into other LCA arenas, especially where it is important to policy makers that a sustainable solution is chosen. This has placed bioenergy in the forefront of LCA development.
